# Patients' values and preferences regarding endoscopic therapy for Barrett's esophagus: A cross‐sectional survey study

**DOI:** 10.1002/deo2.341

**Published:** 2024-02-09

**Authors:** Bashar J. Qumseya, David Estores, Venkata Gorripati, Nanlog Liu, Amira Qumseya

**Affiliations:** ^1^ Division of Gastroenterology, Hepatology and Nutrition University of Florida Gainesville Florida USA; ^2^ Department of Public Health University of Florida Gainesville Florida USA

**Keywords:** Barrett's esophagus, endoscopic eradication therapy, patients’ preferences, patients’ values, willingness to undergo EET

## Abstract

**Introduction:**

Endoscopic eradication therapy (EET) has evolved as a minimally invasive and efficacious option to treat patients with dysplastic Barret's esophagus. We aimed to conduct a cross‐sectional study to assess patient values and preferences on EET.

**Methods:**

All consecutive patients at our clinic and endoscopy center were enrolled between November 2020 and April 2022. The primary outcome was their willingness to undergo EET measured using a validated survey tool. Predictors of this outcome included patient demographics, disease characteristics, procedure types, and physician characteristics. We used a multivariable logistic regression model to assess the association between the primary outcome and its predictors.

**Results:**

A total of 101 consecutive Barret's esophagus patients were surveyed. The median age was 67 years, and 71.3% were males. About 48% (*n =* 48) of the patients had dysplasia, 19% had no dysplasia and others were unsure about the presence or absence of dysplasia. About one‐third (30%, *n =* 30) of patients placed equal values on preventing cancer and avoiding adverse events, and 68% said they were somewhat or definitely willing to undergo EET (ablation and resection). On multivariable logistic regression analysis, the patient value of high emphasis on cancer prevention (odds ratio [OR] 2.9 [1.1–7.6], *p* = 0.0344) and positive relationship with a gastroenterologist (OR 4.7 [1.3–17], *p* = 0.02) were strongly associated with increased willingness to undergo EET.

**Conclusions:**

In this single‐center prospective study of Barret's esophagus patients, the willingness to undergo EET was strongly associated with two predictors: high emphasis on cancer prevention and a positive relationship with the gastroenterologist.

## INTRODUCTION

Esophageal adenocarcinoma (EAC) is a leading cause of cancer mortality in Western countries. Unlike other cancers, the incidence of this disease has been increasing, with possible plateauing in recent years.^1^ Barrett's esophagus (BE) is the primary precursor lesion for EAC.^2^, ^3^ The incidence of BE has also been increasing.^4^ EAC in BE develops through a well‐described cascade of events that start with non‐dysplastic BE (NDBE) that can progress to low‐grade dysplasia (LGD), high‐grade dysplasia (HGD), intramucosal carcinoma (IMC), and continued progression to invasive carcinoma.^5^ Historically, esophagectomy was the only choice in a patient with dysplastic BE. However, in the past two decades, endoscopic eradication therapy (EET) has evolved as a minimally invasive and efficacious option to treat patients with dysplastic BE.^6^

Such therapies include tissue ablation modalities (radiofrequency ablation [RFA], cryotherapy) or resection modalities (endoscopic mucosal resection or dissection),^7^, ^8^, ^9^, ^10^, ^11^, ^12^, ^13^ which have revolutionized the therapeutic approach in these patients. Therefore, national societies recommend EET for patients with HGD, IMC, and even LGD.^6^, ^14^, ^15^ The number of studies on the efficacy and safety of EET has been substantial, leading to the widespread adoption of EET. EET is usually a long‐term commitment needing multiple sessions to achieve the desired results from the patient's standpoint. However, we have scarce data on how patients perceive EET and the factors that may influence their attitudes toward undergoing EET. Previous studies have shown that integrating patients’ preferences and values in the treatment plan can improve patient satisfaction, adherence, and effectiveness.^16^, ^17^ Therefore, a better understanding of how our patients value EET for BE and which factors are associated with improved adherence to EET are critical for gastroenterologists to understand. We aimed to conduct a cross‐sectional survey to assess how patient, disease, procedure, and physician characteristics may shape patient values and preferences regarding EET for BE. Our objective is to analyze factors that shape patient values on various endoscopic management options, thus offering patients healthcare options that are commensurate with their values.

## METHODS

### Study population and outcomes

All adult patients with an existing diagnosis of BE who presented to the gastroenterology clinic or endoscopy suites were eligible for participation. The diagnosis was confirmed based on endoscopy and pathology reports. Consecutive patients at our clinic and endoscopy center were enrolled between December 2021 and May 2022. Patients with an established diagnosis of BE, regardless of dysplasia, were approached about participating in the study. The study was explained to the patients by one of four physicians (two senior faculty members and two advanced GI fellows) using an IRB‐approved consent form. The patient who agreed to participate has to sign the informed consent. Patients were asked to fill out the survey on paper on their own (questions were not read to patients). Patients were allowed to fill out the survey when the doctor left the room. The doctor was blinded to the patient's responses. All discussions on BE took place only after the survey had been completed. The settings of this survey were the clinic (where the majority of patients had no history of EET) and the endoscopy center (where the majority of patients had a history of EET). As this is an impromptu evaluation of the patients’ values they were not provided with standardized education prior to the survey. Patients filled out all surveys on paper forms (Appendix S1).

### Outcomes and predictors

The primary outcomes were the predictors of the willingness to undergo EET, measured on a 5‐point Likert scale. Willingness to undergo EET was defined as a response of somewhat or definitely willingness to undergo ablation and resection modalities (questions 24 and 25). Predictors included:

#### Patients' characteristics

Age (continuous), sex (male vs. female), educational level (college or above vs. high school or below), family history (of BE, EAC, or death related to esophageal cancer)

#### Patients' values and preferences

- Good understanding of risks and benefits of EET: self‐defined understanding as well or very well, question # 17.
- Degree of concern over adverse events (AEs) like stricture, bleeding, and perforation (question # 18)
- Having a value of *high emphasis on cancer prevention*: defined based on responses to question # 19 of the survey: patients who reported that preventing cancer is somewhat or much more important than avoiding adverse outcomes were deemed to have a high emphasis on cancer prevention.

#### Physician factors

- The physician explained the risk of cancer from BE (question # 15): well or very well was considered an adequate explanation.
- Positive relationship with gastroenterologist: positive or very positive response to question # 22.
- The physician explained the risks and benefits of EET (question # 23): well or very well was considered an adequate explanation.
- Clinical settings: The patient is seen in the clinic or the endoscopy center.

#### Procedure or disease factors

- Resection versus ablation
- Degree of dysplasia (dysplasia of any degree vs. none)
- History of EET

The degree of dysplasia was obtained by a review of the patient chart after the survey had been completed.

### Survey validation

The study team designed the survey tool and underwent content, context, and face validation (Appendix). A focus group of two gastroenterologists was asked to comment on the survey. Then, the survey was administered to 10 consecutive patients who were asked to comment on the understandability and appropriateness of the questions. Comments from physicians and patients were used to make changes to the survey used in the study. The final tool consisted of 25 questions answered by patients and three items to be answered by the provider who consented to the patient for the study.

### Statistical analysis

All data from paper surveys were tabulated in Microsoft Excel (Microsoft Corp.) into a password‐protected sheet. None of the data had any patient identifiers. All statistical analysis was done using SAS 9.4 (SAS Institute Inc.). We reported the primary outcome (willingness to undergo EET) as a proportion with 95% confidence intervals (CIs). Univariable regression analyses were used to assess the potential association between outcomes and predictors. Variables with a *p*‐value of <0.1 were included in the multivariable analysis with backward elimination. We used a multivariable logistic regression model to assess the strength of the association between the primary outcome and its predictors while controlling for the confounders. We reported odds ratios (95% CIs and *p*‐values) for each predictor based on the regression model. The institutional review board approved the study at the University of Florida.

## RESULTS

Between December 2021 and May 2022, 104 consecutive patients with BE at the University of Florida were approached to participate in this study. Most patients (97%, *n =* 101) agreed to fill out the survey and formed the study cohort. Of the 101 patients, the median age was 67 years, range of 26–82 years. Of these patients, 71% (*n =* 72) were males, 73% (*n =* 74) had a history of smoking, 92% (*n =* 92) had a history of gastroesophageal reflux disease, 94% (*n =* 95) were on proton pump inhibitors, 56% (*n =* 56) had post‐high school education, 62% (*n =* 63) were retired, and 68% (*n =* 69) were married. Overall, 19% (*n =* 19) reported a family history of BE, 8% (*n =* 8) had a family history of esophageal cancer, and 4% (*n =* 4) had a death in the family related to the EAC. Only 25% (*n =* 25) of patients filled out the survey in the clinic, while the remaining patients took it in the endoscopy suite. Over one‐third (36%, *n =* 36) never had EET. Degree of dysplasia varied from IMC (14%), HGD (39%), LGD (17%), indefinite for dysplasia (IND, 4%), and NDBE (28%). Forty percent of all patients (*n =* 40) had been diagnosed with BE for over three years. The majority of patients had a positive (42%) or very positive (45%) relationship with their gastroenterologist. Further patient characteristics are summarized in Table 1 and Appendix S2.

**TABLE 1:** Baseline characteristics of the 101 patients who participated in the study.

Variable	Frequency	Percentage
Gender		
Male	72	71
Female	29	29
Tobacco use		
No	20	20
Yes	74	73
missing	7	7
Marital status		
Single	12	12
Married	69	68
Divorced	15	15
Widow	5	5
Employment		
Unemployed	3	3
Employed/self	19	19
Home duties	5	5
Retired	63	62
Disabled	11	11
Education		
Not educated	45	45
Educated	56	55
Dysplasia		
LGD	17	17
HGD	20	20
Early cancer	7	7
More than one	4	4
None	19	19
Not sure	27	27
missing	7	7
Treatment History		
RFA	26	26
ESD	6	6
EMR	3	3
None	36	36
Not sure	14	14
missing	16	16
Medication Reflux		
No	6	6
Yes	95	94
Indication		
NDBE	14	14
LGD	4	4
HGD	14	14
IMC	2	2
IND	2	2
Postablation	1	1
Post ESD	2	2
RFA	22	22
Surveillance	12	12
GERD	1	1
Clinic visit	25	25
Missing	2	2

Abbreviations: EMR, endoscopic mucosal resection; ESD, endoscopic submucosal dissection; GERD, gastroesophageal reflux disease; HGD, high‐grade dysplasia; IMC, intramucosal carcinoma; IND, indefinite for dysplasia; LGD, low‐grade dysplasia; NDBE, non‐dysplastic Barrett's esophagus; RFA, radiofrequency ablation.

We asked patients how concerned they were about their BE diagnosis at the time of the first diagnosis and present. Of all patients, 45% (*n =* 45) responded that they were moderately or extremely concerned about the diagnosis when they were first informed of it. This concern appears to be stable over time, with the same number of patients being moderately or extremely concerned at the present time.

Most patients placed a higher value on preventing cancer than avoiding AEs, with 60% (*n =* 60) reporting that preventing cancer is much more important than avoiding AEs. However, about one‐third (30%, *n =* 30) of all patients placed equal values on preventing cancer and avoiding AEs. Similarly, about one‐third of all patients (35%, *n =* 35) reported their understanding of the risks and benefits of treatment to be fair, poor, or very poor. With regards to the specific AEs of EET, patients were more concerned about perforation (37%) and strictures (38%) than bleeding (30%), *p* < 0.001.

### Willingness to undergo EET

Most patients surveyed were willing to undergo EET. Overall, 81% (*n =* 82) of respondents said they were somewhat willing or definitely willing to undergo ablation therapy. On the other hand, 73% (*n =* 74) said they were somewhat or definitely willing to undergo resection therapies. Based on our definitions, 68% (*n =* 69) said they are somewhat or definitely willing to undergo EET (both ablation and resection), while 30% (*n =* 30) said they were neutral, somewhat unwilling, or definitely unwilling (2% [*n =* 2] did not answer).

On univariable analysis, we included two critical measures of patients’ values & preferences that were associated with willingness to undergo EET: high emphasis on cancer prevention (*p* = 0.0325) and a good understanding of the risks versus benefits of EET (0.0066). A positive relationship with a gastroenterologist (defined as positive or very positive, *p* = 0.0271) was also strongly correlated with willingness to undergo EET. There was a trend toward higher willingness to undergo EET based on clinical setting (endoscopy vs. clinic, *p* = 0.0532) and history of EET (*p* = 0.0654), but these met the inclusion criteria for multivariable analysis (p‐value of <0.1). Other predictors, including the degree of dysplasia, did not show a significant association with the primary outcome and are detailed in Table 2.

**TABLE 2:** Univariate analysis for willingness to undergo endoscopic eradication therapy based on several predictor variables.

Predictor	Estimate	Estimate (95% confidence interval)	*p*‐value
*Patient characteristics*			
Age	1.02	(0.98, 1.06)	0.2878
Sex	0.89	(0.35, 2.28)	0.8025
Education	1.38	(0.58, 3.26)	0.4632
Family history	1.39	(0.45, 4.25)	0.5697
*Patient values and preferences*			
Understanding of risks vs benefits	3.50	(1.42, 8.62)	0.0066
Degree of concern about AE	1.24	(0.51, 3.02)	0.6316
High emphasis on cancer prevention	2.68	(1.09, 6.6)	0.0325
*Physician*			
Good explanation of the risk of cancer	2.04	(0.75, 5.53)	0.1629
Positive relationship with physician	4.07	(1.17, 14.15)	0.0271
Discussion of risks and benefits of EET	2.66	(0.95, 7.46)	0.064
Clinical setting (endoscopy vs. clinic)	2.59	(0.99, 6.78)	0.0532
*Disease factor*			
Degree of dysplasia	0.46	(0.18, 1.18)	0.1059
History of EET	2.29	(0.95, 5.52)	0.0654

Abbreviations: AEs, adverse events; EET, endoscopic eradication therapy.

Using backward elimination, we included the following variables into the model: high emphasis on cancer prevention, positive relationship with a gastroenterologist, Clinical setting (endoscopy vs. clinic), and History of EET (yes vs no). On multivariable logistic regression analysis, the patient value of high emphasis on cancer prevention (OR 2.9 [1.1–7.6], *p* = 0.0344) and positive relationship with a gastroenterologist (OR 4.7 [1.3 – 17], *p* = 0.02) was strongly associated with increased willingness to undergo EET (Table 3). All other variables were non‐significant and were dropped from the model. The variable “understanding of risks versus benefits” was collinear with the relationship with a gastroenterologist, so this was excluded from the multivariable logistic regression analysis.

**TABLE 3:** Multivariable analysis of willingness to undergo endoscopic eradication therapy.

Effect	Reference	Estimate	95% confidence interval	*p*‐value
Prevention	Yes	2.9	(1.1, 7.6)	0.0344
Relationship	Yes	4.7	(1.3, 17)	0.02

Abbreviation: EET, endoscopic eradication therapy.

Prevention: high emphasis on cancer prevention (prevention of cancer is much more or somewhat more important than avoiding adverse outcomes).

Relationship: positive or very positive relationship with a gastroenterologist.

## DISCUSSION

In this single‐center, prospective, cross‐sectional study of 101 patients with Barrett's Esophagus, we found that the willingness to undergo EET was strongly associated with two predictors. The first was a patients’ value of high emphasis on cancer prevention, and the second was a positive relationship with the patient's gastroenterologist. Many patients did not clearly understand their management options and placed an equal value on avoiding AEs and preventing cancer. Finally, patients showed more concern about perforation and stricture than bleeding.

Barrett's esophagus is an important clinical entity with increasing incidence. EET offers safe and efficacious options for a patient with dysplastic BE. However, it is a long‐term commitment from the patient; hence, compliance and acceptance of this modality may be primarily influenced by the patients’ values and preferences. Despite the plethora of studies on the efficacy of EET, only a few studies tried to assess how patients perceive EET. Understanding patients’ values and preferences can improve patient satisfaction, adherence, and even effectiveness^16^, ^17^ and is essential to providing patient‐centered high‐quality health care.

Only a few studies addressed the topic of patient preferences in BE. A study by Yachimski et al. assessed the willingness to undergo EET compared to Aspirin.^18^ In this simulated scenario of 81 patients with BE, more participants were more willing to undergo ablation (78%) than to take aspirin (53%). The second study included 50 patients from the University of Chicago and found that 83% of patients were willing to undergo a theoretical EET with a hypothetical success rate of 70%.^19^ The authors found no association between age, education, and gender in a desire to have therapy but recognized that more extensive studies are needed to detect such differences. In a systematic review by Hinojosa‐Lindsey et al.,^20^ five studies were identified exploring patients’ perspectives on surveillance endoscopy in BE. While this study did not address EET, it highlighted several factors that may be associated with increased adherence to surveillance. This included greater trust in physicians (as measured by the Trust in Physician Scale) and health insurance status. Our study adds to this body of literature in several important ways. As far as we know, our study is one of the largest to assess predictors of willingness to undergo EET. Our results indicate that a large subset of the BE population places an equal value on avoiding AE and cancer prevention. This value was associated with willingness to under EET when controlling for other factors. Physicians can easily assess such traits during any clinical interaction. Furthermore, based on the response, physicians need to better explain the risks versus benefits of ETT to prospective patients.

In addition, our study will help inform future clinical practice guidelines on the topic of patients’ values and preferences. However, studies on these topics are scarce. Clinical research often focuses on the efficacy of EET and may not always consider how patients from various backgrounds, educational levels, or socioeconomic statuses may have varying preferences on their treatment options. Most recent guidelines by the ACG recommend EET for a patient with HGD/IMC but also as an option for patients with LGD.^14^ Similarly, the most recent clinical guideline by the American Society for Gastrointestinal Endoscopy (ASGE) suggests EET for LGD.^6^ However, we have a poor understanding of patients' values and preferences concerning EET. The ASGE guidelines on this topic highlighted this problem. Identifying patients' values and preferences in BE was designated as a knowledge gap that needs future research in these guidelines.

Our study highlights some crucial preferences that patients with BE consider when making their decisions. We found that a significant minority of patients placed equal importance on avoiding AE and preventing cancer. This trait strongly predicted willingness to undergo EET on multivariable logistic regression analysis. Such traits may be fundamental in patients with LGD, in whom major guidelines suggest EET, but surveillance is also an acceptable option. Assessing this trait can be relatively easy and could be done during the initial encounter with the patient. Additionally, in patients with a high emphasis on avoiding AE, physicians may need to spend more time and effort detailing the benefits of EET and the potential associated AEs. As seen in our study, furthering patient understanding of the process was associated with increased willingness to undergo EET.

Our survey also shows that a significant minority of patients (one of three) felt they did not clearly understand the risks and benefits. Many patients commented that reading the survey itself gave them a better understanding. Therefore, clinicians may need to spend more time developing written and illustrated patient material to explain the procedures' details to patients. EET can be difficult to understand for the lay patient. We also noted that patients with a positive relationship with their physician were more willing to undergo EET. Therefore, physicians should always strive to build a strong relationship with their patients. Overall, clinicians should ensure that patients make the best decision based on their values.

Our study has obvious limitations. First, this is a single‐center study, thus limiting the generalizability of the findings. Secondly, the sample size may be underpowered to detect some predictors of the willingness to undergo EET. The results of this study should be considered hypothesis‐generating. Also, there was an inherent bias against patients included in the study due to our center being a referral center and that patients are usually motivated toward therapy.

Additionally, we included patients with NDBE in our study even though they do not qualify for EET. We carefully considered this point when we designed our study. While NDBE is not considered for EET, nodular BE (regardless of dysplasia) is considered for EET. Additionally, patients with NDBE may progress to dysplasia on surveillance endoscopy. Therefore, we believe that it was important for us to understand their values and preferences with regard to EET which may be needed in the future. In addition, we considered the history of EET as a predictor of future EET and showed a trend toward higher future willingness to undergo EET, but this was not significant in multivariable regression analysis. On the other hand, we also included patients with IMC, which is an indication for EET, and thus it is important to see how patients with IMC‐valued EET. The question on cancer prevention is assessing a patient attribute: how the patient would value cancer prevention compared to possible AEs. This attribute is important and may be independent of the disease.

Lastly, most of the study took place during the coronavirus disease (COVID) pandemic. We did not specifically address how COVID may have affected patients’ values and preferences with regard to EET. It is plausible that patients who had concerns about COVID infection did not come to their visits and thus, may have not been represented in these results.

Despite the above limitations, this is one of a few studies to assess the values and preferences of patients with BE who may undergo EET. As endoscopists and clinicians, we should use specific patient values and preferences to tailor our interactions with patients to help alleviate some of their concerns and improve the odds of having a successful EET. Our study tries to understand the complex factors that interact to form patients’ values on EET. We hope our results will help physicians provide patient‐centered, individualized counseling and treatments for patients based on their preferences and not on physicians' biases. We hope that our data will encourage future more extensive studies on this vital topic.

## CONFLICT OF INTEREST STATEMENT

Dr. Qumseya is a consultant for Medtronic, Assertio Management, and Endogastric Solutions, and received travel reimbursement from Castle Biosciences.

## Supporting information


**Appendix S1** Validated survey tool.

**Appendix S2** Details and distribution of responses to key questions in the survey tool.
