# Supplementary material for: Patients' values and preferences regarding endoscopic therapy for Barrett's esophagus: A cross‐sectional survey study
Source: DEN Open. 2024 Feb 9;4(1):e341. doi: 10.1002/deo2.341 (PMC10858320; doi:10.1002/deo2.341)
Supplement: Supplementary file 1 — Appendix S1 Validated survey tool. [file DEO2-4-e341-s001.docx]

| _1_□Male | _2_□Female | _3_□No answer |
| --- | --- | --- |

1. How old are you? _________Years 2. Biological gender is:
2. Do you have medical insurance or healthcare coverage? _Yes No_
3. Do you use, or have you ever-used, tobacco products? _Yes No_

| _0_□Single | _1_□ Married | _2_□ Divorced | _3_□ Widow |
| --- | --- | --- | --- |

1. What is your marital status?

| _0_□Unemployed | _1_□Employed/Self-employed | _2_□ Home duties | _3_□Retired | _4_□ Disabled |
| --- | --- | --- | --- | --- |

1. What is your employment status?

| _0_□ Never attended school | _1_□ Elementary/middle school | _2_□ High School |
| --- | --- | --- |
| _3_□ Two-year diploma | _4_□ College/University (4 years) | _5_□ Masters or higher |

1. What is your highest level of education?

1. Do you have a history of heartburn or acid reflux?
2. Are currently on any medications for acid reflux?

| _0_□No | _1_□Yes |
| --- | --- |
| _0_□No | _1_□Yes |

1. How long ago to you learn that you have Barrett’s Esophagus (BE)?

| < 1 year | 1 to 2 years | 3 – 5 years | >5 years |
| --- | --- | --- | --- |

1. When you were informed that you have BE, how concerned were you about it?

| _1_□ Not at all | _2_□ A little | _3_□ somewhat | _4_□ Moderately | _5_□ Extremely |
| --- | --- | --- | --- | --- |

1. At the current time, how concerned are you about BE?

| _1_□ Not at all | _2_□ A little | _3_□ somewhat | _4_□ Moderately | _5_□ Extremely |
| --- | --- | --- | --- | --- |

1. Do you have a parent or sibling who has one or more of the following:

A) Barrett’s esophagus B) Cancer of the esophagus C) Death related to cancer of the esophagus

| _0_□No | _1_□Yes |
| --- | --- |

| _0_□No | _1_□Yes |
| --- | --- |

| _0_□No | _1_□Yes |
| --- | --- |

1. Dysplasia is a term used to describe precancerous changes the Barrett’s Esophagus. Have you even been told that you have dysplasia?

| _0_□ Low-grade dysplasia | _1_□ High-grade dysplasia | _2_□ Early cancer | _4_□ More than one of the above | _5_□ None of the above | _6_□ Not sure |
| --- | --- | --- | --- | --- | --- |

1. Do you feel that your physician has adequately explained to you the risk of cancer from Barrett’s esophagus?

| _1_□ Very poorly | _2_□ Poorly | _3_□ Fairly | _4_□ Well | _5_□Very well |
| --- | --- | --- | --- | --- |

1. Have you ever received any of the following treatments for Barrett’s esophagus (choose all that apply)?

| _1_□ Radiofrequency ablation (RFA) | _2_□ Cryotherapy | _3_□ Photodynamic therapy | _4_□ Endoscopic submucosal dissection (ESD) |
| --- | --- | --- | --- |
| _5_□ Endoscopic mucosal resection (EMR) | _6_□ None of the above | _7_□ Not Sure |  |

1. Do you feel that you have a good understanding of the risks and benefits of Barrett’s treatment options?

| _1_□ Very poorly | _2_□ Poorly | _3_□ Fairly | _4_□ Well | _5_□Very well |
| --- | --- | --- | --- | --- |

1. The endoscopic treatments mentioned above have potential adverse outcomes in about 9% of all patients. Those including narrowing in the esophagus (stricture), bleeding requiring blood transfusion/admission to the hospital (2%), and a break in the lining for the esophagus (perforation <1%). How concerned are you about having one or more of those outcomes?

| Stricture: narrowing in the esophagus | _1_□ Not at all | _2_□ A little | _3_□ somewhat | _4_□ Moderately | _5_□ Extremely |
| --- | --- | --- | --- | --- | --- |
| Bleeding: requiring blood transfusion | _1_□ Not at all | _2_□ A little | _3_□ somewhat | _4_□ Moderately | _5_□ Extremely |
| Perforation: break in the lining of the esophagus | _1_□ Not at all | _2_□ A little | _3_□ somewhat | _4_□ Moderately | _5_□ Extremely |

1. In treating BE, there is a trade-off between trying to prevent/cure cancer and avoiding adverse outcomes. Which of the following is most important to you?

| _1_□ Preventing cancer is much more important to me than avoiding adverse outcomes |
| --- |
| _2_□ Preventing cancer is somewhat more important than avoiding adverse outcomes |
| _3_□ Preventing cancer is equally as important to avoiding adverse outcomes |
| _4_□ Avoiding adverse outcomes is somewhat more important than preventing cancer |
| _5_□ Avoiding adverse outcomes is much important than preventing cancer |

1. The risk of developing cancer in Barrett’s esophagus is greatly dependent on the degree of dysplasia. The risk cancer in patients with no dysplasia is very low. For patients with low-grade dysplasia, the lifetime risk is about 6% with no treatment and 1.2% if treated. For high-grade dysplasia, the life-long risk of developing cancer is 27% if not treated and 7% if treated. How **acceptable** is treatment for you if you have:

| No dysplasia | _1_□ Not at all | _2_□ A little | _3_□ somewhat | _4_□ Moderately | _5_□ Extremely |
| --- | --- | --- | --- | --- | --- |
| Low Grade dysplasia | _1_□ Not at all | _2_□ A little | _3_□ somewhat | _4_□ Moderately | _5_□ Extremely |
| High Grade dysplasia | _1_□ Not at all | _2_□ A little | _3_□ somewhat | _4_□ Moderately | _5_□ Extremely |

| < 1 year | 1 to 2 years | 3 – 5 years | >5 years |
| --- | --- | --- | --- |

1. How long have you known your primary gastroenterologist?
2. How would you describe your relationship with this gastroenterologist?

| _1_□ Very negative | _2_□ Negative | _3_□ Neutral | _4_□ Positive | _5_□ Very positive |
| --- | --- | --- | --- | --- |

1. How well did you gastroenterologist explain to you the risks and benefits of treatment for BE?

| _1_□ Very poorly | _2_□ poorly | _3_□ neutral | _4_□ well | _5_□ very well |
| --- | --- | --- | --- | --- |

1. Ablation therapies like radiofrequency ablation (RFA), cryotherapy, and photo-dynamic therapy (PDT), have a small risk of bleeding and a very small risk of breaking the lining of your esophagus. Based on the above, how willing are you to go ablation therapies?

| _1_□ Definitely not willing | _2_□ somewhat not willing | _3_□ neutral | _4_□ somewhat willing | _5_□ definitely willing |
| --- | --- | --- | --- | --- |

1. Resection modalities, like endoscopic mucosal resection/dissection, involve removing the inner inning of an area in your esophagus. These techniques care cure early cancer and give a definite diagnosis of more advanced cancers. However, there is a risk of bleeding in about 5% and risk of breaking the lining of the esophagus (which could require surgery to fix it) in about 1%. Based on the above, how willing are you to go resection therapies if needed?

| _1_□ Definitely not willing | _2_□ somewhat not willing | _3_□ neutral | _4_□ somewhat willing | _5_□ definitely willing |
| --- | --- | --- | --- | --- |

Thank you for filling out our survey!

To be filled by physician after patient has completed form.

Date:

Indication for procedure or clinic visit:

Highest Grade of dysplasia:

Number of endoscopies in the last year:
