# Supplementary material for: Patients' values and preferences regarding endoscopic therapy for Barrett's esophagus: A cross‐sectional survey study
Source: DEN Open. 2024 Feb 9;4(1):e341. doi: 10.1002/deo2.341 (PMC10858320; doi:10.1002/deo2.341)
Supplement: Supplementary file 2 — Appendix S2 Details and distribution of responses to key questions in the survey tool. [file DEO2-4-e341-s002.docx]

**Appendix 2**: break down of responses to questions in the survey

| **Question Number** | **Response** | **Number of respondents** | **Percentage** |
| --- | --- | --- | --- |
| **10** | 1. How long ago to you learn that you have Barrett’s Esophagus (BE)? | | |
|  | 1 | 14 | 14 |
|  | 1-2 years | 16 | 16 |
|  | 3-5 years | 22 | 22 |
|  | >5 | 49 | 49 |
| **11** | When you were informed that you have BE, how concerned were you about it? | | |
|  | Not at all | 4 | 4 |
|  | A little | 11 | 11 |
|  | Some what | 24 | 24 |
|  | moderately | 32 | 32 |
|  | extremely | 29 | 29 |
| **12** | At the current time, how concerned are you about BE? | | |
|  | Not at all | 4 | 4 |
|  | A little | 13 | 13 |
|  | Some what | 24 | 24 |
|  | moderately | 30 | 30 |
|  | extremely | 30 | 30 |
| **13** | 1. Do you have a parent or sibling who has one or more of the following? | | |
|  | 1. Barrett’s Esophagus | | |
|  | No | 81 | 81 |
|  | Yes | 19 | 19 |
|  | missing | 1 | 1 |
|  | 1. Cancer of the esophagus | | |
|  | missing | 3 | 3 |
|  | No | 90 | 90 |
|  | Yes | 8 | 8 |
|  | 1. Death related to cancer of the esophagus | | |
|  | missing | 5 | 5 |
|  | No | 92 | 92 |
|  | Yes | 4 | 4 |
| **14** | Dysplasia is a term used to describe precancerous changes the Barrett’s Esophagus. Have you even been told that you have dysplasia? | | |
|  | missing | 7 | 7 |
|  | LGD | 17 | 17 |
|  | HGD | 20 | 20 |
|  | Early cancer | 7 | 7 |
|  | More than one | 4 | 4 |
|  | None | 19 | 19 |
|  | Not sure | 27 | 27 |
| **16** | Have you ever received any of the following treatments for Barrett’s esophagus (choose all that apply)? | | |
|  | missing | 16 | 16 |
|  | RFA | 26 | 26 |
|  | ESD | 6 | 6 |
|  | EMR | 3 | 3 |
|  | None | 36 | 36 |
|  | Not sure | 14 | 14 |
| **18** | 1. The endoscopic treatments mentioned above have potential adverse outcomes in about 9% of all patients. Those including narrowing in the esophagus (stricture), bleeding requiring blood transfusion/admission to the hospital (2%), and a break in the lining for the esophagus (perforation <1%). How concerned are you about having one or more of those outcomes? | | |
| **Stricture** | Missing | 2 | 2 |
|  | Not at all | 10 | 10 |
|  | A little | 26 | 26 |
|  | Somewhat | 24 | 24 |
|  | Moderately | 31 | 31 |
|  | Extremely | 7 | 7 |
| **Bleeding** | Missing | 0 | 0 |
|  | Not at all | 17 | 17 |
|  | A little | 22 | 22 |
|  | Somewhat | 29 | 29 |
|  | Moderately | 22 | 22 |
|  | Extremely | 10 | 10 |
| **Perforation** | Missing | 2 | 2 |
|  | Not at all | 17 | 17 |
|  | A little | 22 | 22 |
|  | Somewhat | 29 | 29 |
|  | Moderately | 22 | 22 |
|  | Extremely | 10 | 10 |
| **19** | 1. In treating BE, there is a trade-off between trying to prevent/cure cancer and avoiding adverse outcomes. Which of the following is most important to you? | | |
|  | missing | 1 | 0.99 |
|  | Much more | 60 | 59.41 |
|  | Somewhat | 8 | 7.92 |
|  | equally | 30 | 29.70 |
|  | Avoiding somewhat | 1 | 0.99 |
|  | Avoiding Much | 1 | 0.99 |
| **20** | How **acceptable** is treatment for you if you have? | | |
| **No dysplasia** | missing | 15 | 14.85 |
|  | Not at all | 19 | 18.81 |
|  | A little | 17 | 16.83 |
|  | Some what | 21 | 20.79 |
|  | moderately | 6 | 5.94 |
|  | extremely | 23 | 22.77 |
| **LGD** | missing | 15 | 14.85 |
|  | Not at all | 5 | 4.95 |
|  | A little | 12 | 11.88 |
|  | Some what | 19 | 18.81 |
|  | moderately | 18 | 17.82 |
|  | extremely | 32 | 31.68 |
| **HGD** | missing | 12 | 11.88 |
|  | Not at all | 2 | 2 |
|  | A little | 6 | 6 |
|  | Some what | 11 | 11 |
|  | moderately | 15 | 15 |
|  | extremely | 55 | 55 |
| **21** | How long have you known your primary gastroenterologist? | | |
|  | Missing | 4 | 4 |
|  | 1 | 41 | 41 |
|  | 1-2 years | 16 | 16 |
|  | 3-5 years | 12 | 12 |
|  | >5 | 27 | 27 |
|  | 5 | 1 | 1 |
| **22** | 1. How would you describe your relationship with this gastroenterologist? | | |
|  | missing | 2 | 2 |
|  | Negative | 3 | 3 |
|  | Neutral | 9 | 9 |
|  | Positive | 42 | 42 |
|  | very positive | 45 | 45 |
| **23** | 1. How well did you gastroenterologist explain to you the risks and benefits of treatment for BE? | | |
|  | missing | 2 | 2 |
|  | Very negative | 4 | 4 |
|  | Negative | 3 | 3 |
|  | Neutral | 12 | 12 |
|  | Positive | 31 | 31 |
|  | very positive | 49 | 49 |
| **24** | 1. Ablation therapies like radiofrequency ablation (RFA), cryotherapy, and photo-dynamic therapy (PDT), have a small risk of bleeding and a very small risk of breaking the lining of your esophagus. Based on the above, how willing are you to go ablation therapies? | | |
|  | Definitely not willing | 0 | 0 |
|  | somewhat not willing | 2 | 2 |
|  | neutral | 16 | 16 |
|  | somewhat willing | 19 | 19 |
|  | definitely willing | 63 | 63 |
| **25** | 1. Resection modalities, like endoscopic mucosal resection/dissection, involve removing the inner inning of an area in your esophagus. These techniques care cure early cancer and give a definite diagnosis of more advanced cancers. However, there is a risk of bleeding in about 5% and risk of breaking the lining of the esophagus (which could require surgery to fix it) in about 1%. Based on the above, how willing are you to go resection therapies if needed? | | |
|  | Missing | 1 | 1 |
|  | Definitely not willing | 0 | 0 |
|  | somewhat not willing | 3 | 3 |
|  | neutral | 22 | 22 |
|  | somewhat willing | 26 | 26 |
|  | definitely willing | 48 | 48 |
